# The reemergence of long-term potentiation in aged Alzheimer’s disease mouse model

**DOI:** 10.1038/srep29152

**Published:** 2016-07-05

**Authors:** Seonghoo Huh, Soo-Ji Baek, Kyung-Hwa Lee, Daniel J. Whitcomb, Jihoon Jo, Seong-Min Choi, Dong Hyun Kim, Man-Seok Park, Kun Ho Lee, Byeong C. Kim

**Affiliations:** 1 Chonnam-Bristol Frontier Laboratory, Biomedical Research Institute, Chonnam National University Hospital, Gwangju 61469, Republic of Korea; 2 Department of Biomedical Sciences, Chonnam National University Medical School, Gwangju 61469, Republic of Korea; 3 Department of Pathology, Chonnam National University Medical School, Gwangju 61469, Republic of Korea; 4 Henry Wellcome Laboratories for Integrative Neuroscience and Endocrinology, School of Clinical Sciences, Faculty of Health Sciences, University of Bristol, Whitson Street, Bristol BS1 3NY, UK; 5 Department of Neurology, Chonnam National University Medical School, Gwangju 61469, Republic of Korea; 6 Department of Medicinal Biotechnology, College of Health Sciences and Dong-A Anti-aging Research Center, Dong-A University, Busan 49315, Republic of Korea; 7 National Research Center for Dementia, Gwangju 61452, Republic of Korea

## Abstract

Mouse models of Alzheimer’s disease (AD) have been developed to study the pathophysiology of amyloid β protein (Aβ) toxicity, which is thought to cause severe clinical symptoms such as memory impairment in AD patients. However, inconsistencies exist between studies using these animal models, specifically in terms of the effects on synaptic plasticity, a major cellular model of learning and memory. Whereas some studies find impairments in plasticity in these models, others do not. We show that long-term potentiation (LTP), in the CA1 region of hippocampal slices from this mouse, is impared at Tg2576 adult 6–7 months old. However, LTP is inducible again in slices taken from Tg2576 aged 14–19 months old. In the aged Tg2576, we found that the percentage of parvalbumin (PV)-expressing interneurons in hippocampal CA1-3 region is significantly decreased, and LTP inhibition or reversal mediated by NRG1/ErbB signaling, which requires ErbB4 receptors in PV interneurons, is impaired. Inhibition of ErbB receptor kinase in adult Tg2576 restores LTP but impairs depotentiation as shown in aged Tg2576. Our study suggests that hippocampal LTP reemerges in aged Tg2576. However, this reemerged LTP is an insuppressible form due to impaired NRG1/ErbB signaling, possibly through the loss of PV interneurons.

Progressive loss of memory function and the accumulation of amyloid β protein (Aβ) in the brain are key characteristics of Alzheimer’s disease (AD) pathology^1^,^2^. Previous findings have demonstrated that synaptic plasticity, regarded as the cellular basis for learning and memory^3^, is vulnerable to exposure to high levels of Aβ; hippocampal long-term potentiation (LTP) is impaired following exogenous exposure to soluble oligomeric Aβ^4^,^5^,^6^. To try to understand how memory function in AD becomes dysregulated, a vast number of studies have looked to determine the mechanisms underlying the Aβ-mediated impairment of synaptic plasticity.

To closely model AD pathology, transgenic mice carrying mutant amyloid precursor protein (APP), which causes excessive production of Aβ in the brain, have been developed, and are used to investigate the pathophysiology of Aβ toxicity^7^. However, inconsistencies remain in the pathological readouts from such animals; whether LTP is inhibited or normal in these mouse models remains unclear, as notably reported in papers using one of the most frequently used transgenic mice harboring the Swedish mutation in APP (APP_695_SWE)^8^,^9^. Here, whilst some studies report inhibition of LTP in the APP transgenic mouse^10^,^11^,^12^, others find no inhibition of LTP between 3 months and 12 months of age in these AD model mice^13^,^14^,^15^,^16^. An important aspect to consider in relation to this is the diverse ages of the animals used in these studies (ranging from 3 to 18 months of age). Here, some studies have reported a relationship between abnormal synaptic plasticity and the age of the APP transgenic mouse used, with young animals displaying profound LTP impairments that are not present in the older animals^15^,^17^. The reason for this apparent age-dependent effect, however, is unknown.

One explanation for these age-dependent effects could relate to the interneuronal control of pyramidal neurons. Most GABAergic interneurons in the hippocampal CA1 region innervate their synapses onto pyramidal cell dendrites where neuronal computation, such as modulation, integration of synaptic inputs and expression of synaptic plasticity, mainly occurs^18^,^19^,^20^. Recently, it was shown that loss of GABAergic interneurons leads to an enhanced LTP in the hippocampal CA1 region^21^. Interestingly, a significant decrease in the number of GABAergic interneurons was observed in the hippocampus of the AD mouse model and indeed in AD patients^22^,^23^. Furthermore, enhanced LTP was accompanied with reduced numbers of interneurons in the dentate gyrus of the mouse model of both amyloidosis and tauopathy^24^. One possible explanation, therefore, is that the age-dependent loss of GABAergic interneurons in the transgenic mice actually serves to facilitate the induction of LTP in the hippocampal CA1 region.

Both ErbB receptor kinases in GABAergic interneurons and the endogenous ligand neuregulin 1 (NRG1), a neurotropic factor implicated in neural development, neurotransmission and synaptic plasticity^25^, have been variously shown to regulate hippocampal LTP: (1) neutralization of endogenous NRG1 in the hippocampus enhances the magnitude of hippocampal LTP at CA1 region^26^, whereas addition of exogenous NRG1 suppresses the induction of LTP^27^,^28^; (2) inhibition of ErbB4 kinase increases the magnitude of hippocampal LTP, an effect similarly observed in *ErbB4* knockout mice^29^,^30^; (3) ErbB4 is selectively expressed in interneurons but not in pyramidal neurons^31^, and *ErbB4* deletion in parvalbumin (PV) interneurons, the major type of GABAergic interneurons in hippocampus^32^,^33^, completely blocks the LTP regulation induced by NRG1^26^,^30^. Critically, NRG1 and ErbB4 distribution was found to be altered in the brains spotted with neuritic plaques in the mouse model of AD and in AD patients^34^. We therefore hypothesized that the age-dependent dysregulation of hippocampal LTP in the APP transgenic mouse was underpinned by the Aβ-mediated impairment of NRG1/ErbB signaling, leading to the facilitation of LTP. Here we demonstrate a possible link between a loss of interneurons, NRG1/ErbB signaling dysregulation and changes in synaptic plasticity in a mouse model of AD.

## Results

### The reemergence of LTP in the hippocampus of aged Tg2576

**Figure 1:**
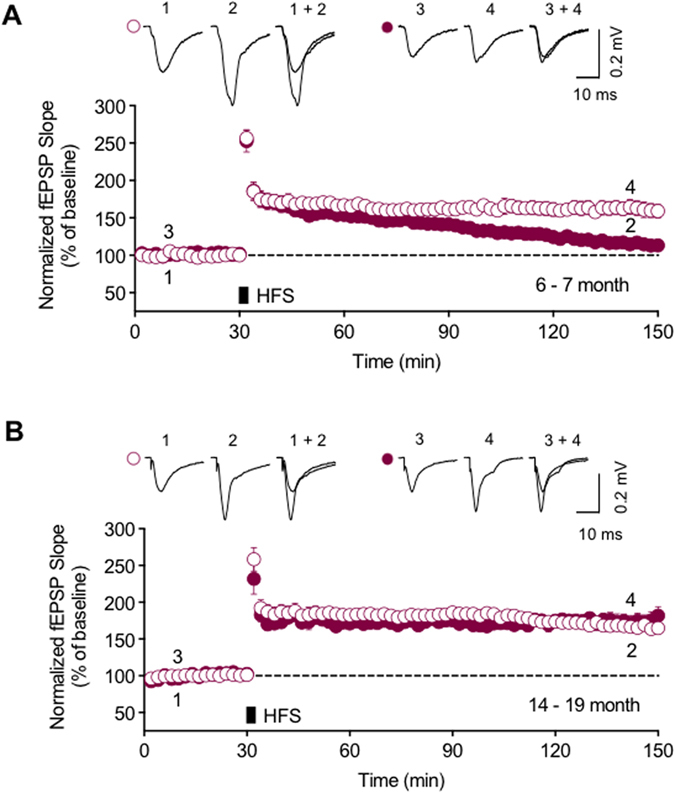
The reemergence of LTP in the hippocampus of aged Tg2576. (**A**) Applying two trains of tetanus stimuli (10 Hz in  s, 3 s inter-train interval) to the Schaffer collateral pathway evoked LTP in the hippocampal CA1 region in 6–7 months old WT (open circle, *n* = 8) but did not in Tg2576, where the fEPSP was gradually decreased to the baseline level 2 hours after LTP induction by HFS (closed circle, *n* = 8). (**B**) In 14–19 months old animals, LTP was observed in both WT (open circle, *n* = 7) and Tg2576 (closed circle, *n* = 7) mice, and their increased fEPSP levels were not significantly different (*P* > 0.05). Error bars represent standard error of the mean (SEM). fEPSP = field excitatory postsynaptic potential.

To examine whether LTP in Tg2576 mice is altered in an age-dependent manner, we compared LTP in two different age groups, 6–7 months old (adult) and 14–19 months old (aged), in the hippocampal CA1 region. Tg2576 is a well-documented mouse model of AD showing high levels of Aβ and memory impairment^8^,^10^, and soluble Aβ in the brains of these transgenic animals begins to be significantly accumulated at around 6 months of age after a non-accumulation phase from birth^11^,^35^. Increased fEPSP induced by high frequency stimulation (HFS) at the Schaffer collateral pathway was maintained for 2 hours in adult littermate WT, but returned to baseline levels in adult Tg2576 (Tg: 113.2 ± 7.4% of baseline, *n* = 8, closed circle; WT: 159.1 ± 9.4%, *n* = 8, open circle, *P* < 0.01 Fig. 1A), which is consistent with reports describing impaired LTP in Tg2576 of similar ages^12^,^15^. In contrast, the increased fEPSPs were maintained until 2 hours after HFS application in aged Tg2576, and there were no significant differences in the levels of the potentiated fEPSPs between the aged Tg2576 and littermate WT mice (Tg: 181.7 ± 12.0%, *n* = 7, closed circle; WT: 164.8 ± 7.2%, *n* = 7, open circle, *P* > 0.05, Fig. 1B). Interestingly, the observed time period for LTP inhibition and reappearance in Tg2576 mice is very similar to a previous report which pointed out that soluble Aβ levels in the brain was not correlated with the LTP deficit in aged animals^15^. Clearly, then, there is an interesting disparity in impaired synaptic plasticity in these AD mouse models; whilst LTP is inhibited at early ages after soluble Aβ accumulation begins, it reemerges at older ages.

### Decreased percentage of PV interneurons in hippocampal CA region of aged Tg2576

**Figure 2:**
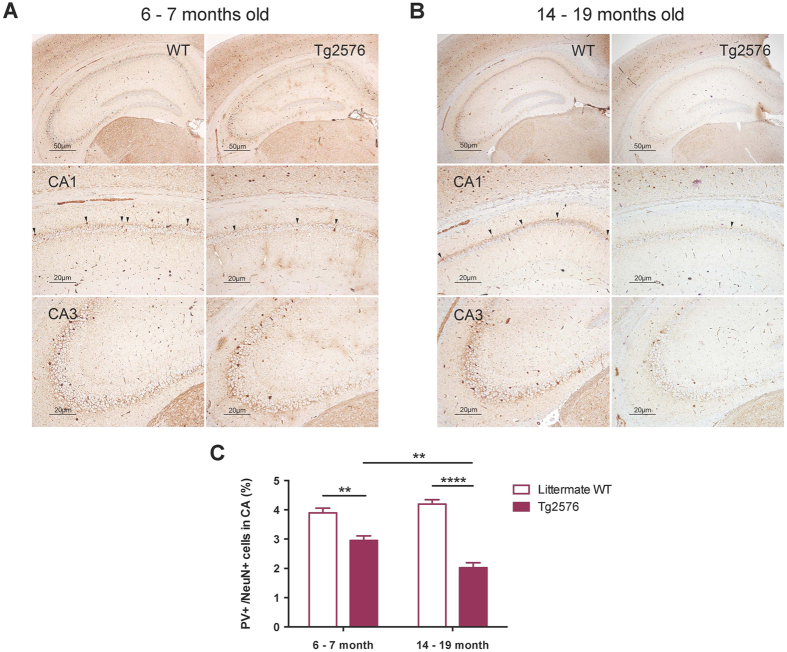
The percentage of parvalbumin (PV) interneurons is reduced in aged Tg2576. (A) Representative PV immunohistochemistry stains in the hippocampus of a 6–7 months old (adult) WT (n = 4) and Tg2576 (n = 4) mouse. PV-positive interneurons are shown in the hippocampus at a low magnification (original magnification, ×40), and at a closer distance from the CA1 (original magnification, ×100, pointed by arrowheads) and CA3 (original magnification, ×100). (**B**) Representative PV immunohistochemistry stains in the hippocampus of a 14–19 months old (aged) WT (n = 6) and Tg2576 (n = 6) mouse. (**C**) The ratio of PV-positive cells to NeuN-positive cells (data not shown) in aged Tg2576 is significantly lower than in adult Tg2576 (**P < 0.01, ****P < 0.0001).

One possible explanation for the LTP reemergence in aged Tg2576 would be the decreased density of inhibitory interneurons in hippocampus, which could conceivably contribute to an enhancement of excitatory signaling and the facilitation of LTP. To test this hypothesis, we specifically measured the percentage of PV interneurons in hippocampal CA1-3 region. PV interneurons constitute a major proportion of GABAergic interneurons in the hippocampus^32^,^33^. Further, PV interneurons are critical for the NRG1/ErbB4-mediated down-regulation of LTP expression at the Schaffer collateral-CA1 synapse^26^,^30^. PV-immunoreactive (IR) neurons were observed in hippocampus of both the adult and aged Tg2576 (Fig. 2A,B). To calculate the percentage of PV interneurons, NeuN and PV-IR neurons were counted in whole CA subfields, and the ratio of PV-IR to NeuN-IR cells was estimated. We found that the proportion of PV interneurons to NeuN-positive cells was significantly decreased in aged Tg2576 compared with adult Tg2576 (Fig. 2C). Importantly, this reduction was not solely due to the aging effect, since there was no significant difference in the percentage of the neurons between adult and aged littermate WT. Interestingly, it was recently shown that loss of PV interneurons is associated with enhanced LTP in a mouse model of multiple sclerosis^36^, suggesting a strong possibility of this neuronal loss enhancing LTP in aged Tg2576.

### NRG1-induced LTP inhibition and depotentiation are impaired in aged Tg2576

**Figure 3:**
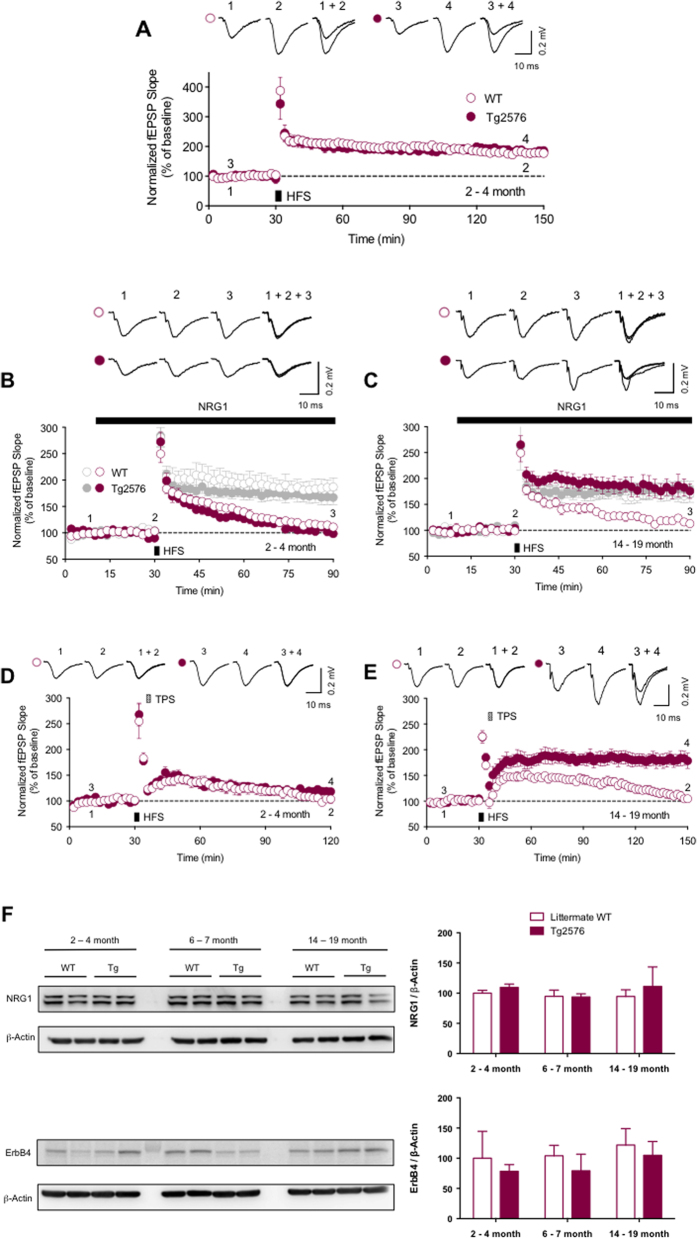
NRG1-induced LTP inhibition and TPS-induced depotentiation are impaired in aged Tg2576. (**A**) LTP was readily induced in both 2–4 months old WT (open circle, *n* = 7) and Tg2576 (closed circle, *n* = 7) mice. (**B**) Perfusion of  nM NRG1 peptide during the period indicated by the heavy line suppressed LTP in both 2–4 months old WT (maroon open circle, *n* = 6), and Tg2576 (maroon closed circle, *n* = 6). (**C**) NRG1 perfusion also suppressed LTP in 14–19 months old WT (maroon open circle, *n* = 6), but did not in Tg2576 (maroon closed circle, *n* = 6) (**D**) Applying theta pulse stimulation (TPS, 100 pulses with  Hz) 5 minutes after HFS reversed LTP in both 2–4 months old WT (open circle, *n* = 6) and Tg2576 (closed circle, *n* = 7). (**E**) TPS also reversed LTP in 14–19 months old WT (open circle, *n* = 6), but did not in Tg2576 (closed circle, *n* = 6). (**F**) In both Tg2576 and WT mice, the expression of NRG1 and ErbB4 in the hippocampus was not significantly different among 3 aged groups (*P* > 0.05). However, this data showed that the expression of ErbB4 tended to decrease in Tg2576 (closed bar, *n* = 5) in comparison with littermate WT (open bar, *n* = 5) at each aged group. Two-way ANOVA and Turkey’s multiple comparison tests were used for the statistical analysis of this data. Error bars represent SEM. fEPSP = field excitatory postsynaptic potential.

We next tested whether the regulation of synaptic plasticity by NRG1/ErbB signaling is intact in aged Tg2576. Previous studies demonstrated that perfusion of hippocampal slices with recombinant NRG1 peptide inhibits the induction of hippocampal LTP by activating ErbB4 receptor kinase in PV interneurons^26^,^30^. We first measured hippocampal LTP in 2–4 months old (young) Tg2576, in which Aβ is produced only marginally in the brain^35^, and littermate WT. We found that LTP was readily induced in both age groups (Tg: 185.5 ± 11.2%, *n* = 7, closed circle; WT: 177.4 ± 10.3%, *n* = 7, open circle, *P* > 0.05, Fig. 3A). We next investigated the NRG1-induced LTP inhibition using those mice, by measuring fEPSP in hippocampal slices perfused with 1 nM NRG1 peptide, stimulated with HFS 20 minutes after onset of NRG1 perfusion. The NRG1 perfusion readily suppressed the induction of LTP in both young Tg2576 and littermate WT (Tg vehicle: 166.9 ± 13.6%, *n* = 7, grey closed circle; WT vehicle: 187.0 ± 17.0%, *n* = 6, grey open circle; Tg NRG1: 98.4 ± 6.4%, *n* = 6, maroon closed circle, *P* < 0.01 compared with Tg vehicle; WT NRG1: 111.0 ± 3.7%, *n* = 6, maroon open circle, *P* < 0.01 compared with WT vehicle, Fig. 3B). We then tested the integrity of NRG1-induced LTP inhibition in aged Tg2576 and littermate WT. Interestingly, we found that NRG1 perfusion inhibited LTP induction in aged WT, but did not in aged Tg2576 (Tg vehicle: 179.0 ± 15.3%, *n* = 5, grey closed circle; WT vehicle: 170.2 ± 16.1%, *n* = 6, grey open circle; Tg NRG1: 175.6 ± 14.1%, *n* = 6, maroon closed circle, *P* > 0.05 compared with Tg vehicle; WT NRG1: 112.7 ± 4.4%, *n* = 6, maroon open circle, *P* < 0.05 compared with WT vehicle, Fig. 3C). These data suggest that an NRG1/ErbB-mediated signaling pathway that can suppress LTP does not function normally in aged Tg2576 in which LTP reappears after the deficit period at adult age.

NRG1/ErbB signaling is known to be involved in depotentiation, another form of synaptic plasticity where LTP can be reversed by a specific form of synaptic activity induced within a few minutes following LTP induction^37^,^38^. It has been shown that the application of NRG1 peptide or theta-pulse stimulation (TPS), a low frequency stimulation that is widely used to induce depotentiation^37^, reverses previously induced hippocampal LTP, and ErbB4 in PV interneurons is crucial for this effect^30^,^39^. To examine whether depotentiation is intact in both young and aged Tg2576, we applied TPS to the Schaffer collateral pathway in the hippocampus 5 minutes after HFS application. We found that potentiated fEPSPs by HFS application returned to the baseline at 1.5 hours after applying TPS in both young Tg2576 and littermate WT, indicative of a depotentiation of synapses (Tg: 118.3 ± 7.0%, *n* = 7, closed circle; WT: 103.8 ± 8.3%, *n* = 6, open circle, *P* > 0.05, Fig. 3D). LTP was also readily depotentiated by the application of TPS in aged littermate WT, but remained at an elevated level 2 hours after applying TPS in aged Tg2576 (Tg: 178.4 ± 13.7%, *n* = 6, closed circle; WT: 104.7 ± 5.0%, *n* = 6, open circle, *P* < 0.01, Fig. 3E). The absence of depotentiation in slices from aged Tg2576 animals is consistent with NRG1/ErbB signaling being impaired in aged Tg2576, and suggests that synaptic function in aged Tg2576 is abnormal in spite of the reemergence of LTP. We also observed that, in both Tg2576 and WT mice, the expression of NRG1 and ErbB4 in the hippocampus was not significantly different among 3 age groups (*P* > 0.05). However, this data showed that the expression of ErbB4 tended to decrease in Tg2576 in comparison with littermate WT (Tg: 78 ~ 82%, n = 5, closed bar, *P* > 0.05, Fig. 3F).

### Inhibition of ErbB receptor kinase in adult Tg2576 can mimic the changes of the synaptic plasticity in aged Tg2576

**Figure 4:**
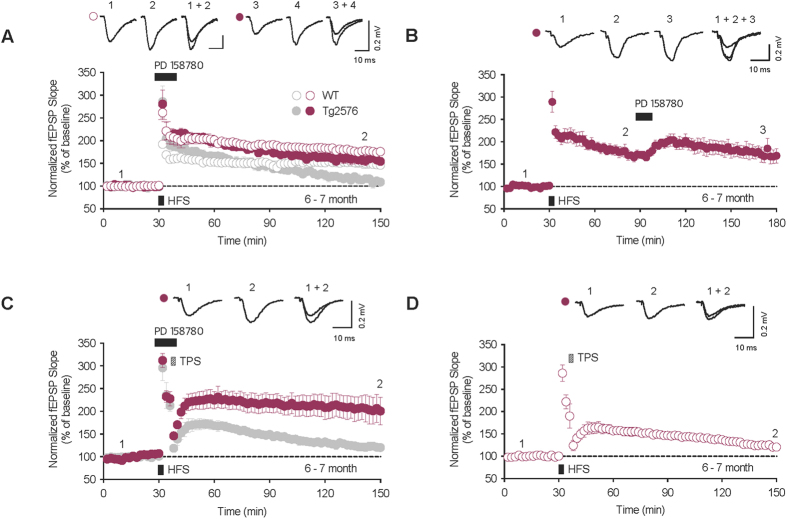
ErbB inhibitor restores LTP but blocks depotentiation in adult Tg2576. (**A**) Perfusion of 10 μM PD158780, an inhibitor of ErbB, for 10 minutes starting two minutes before HFS, as indicated by the heavy line, restored LTP in 6–7 months old Tg2576 (maroon closed circle, *n* = 6), and enhanced LTP in 6–7 months old wild-type (maroon open circle, n = 6). (**B**) Perfusion of PD158780 for 10 minutes starting an hour after HFS increased fEPSP slope, which was continuously decreased just before, and the re-potentiated fEPSP slope was maintained for another two hours. (closed circle, *n* = 6) (**C**) TPS did not reverse LTP in the hippocampal slices from 6–7 months old Tg2576 perfused with PD158780 (closed circle, *n* = 6). (**D**) TPS readily reversed LTP in 6–7 months old WT slices (open circle, *n* = 5). Error bars represent SEM. fEPSP = field excitatory postsynaptic potential.

If the impaired NRG1/ErbB signaling is actually correlated with the reemerged LTP in aged Tg2576 mice, disrupting NRG1/ErbB signaling in adult Tg2576 might induce similar synaptic characteristics to those observed in aged Tg2576: normal LTP but impaired depotentiation. We therefore investigated the effect of blocking the ErbB4 receptor on synaptic plasticity in adult Tg2576. To do this, we first perfused hippocampal slices from adult Tg2576 with 10 μM PD158780, an inhibitor of ErbB receptor kinase, for 10 minutes starting 2 minutes before HFS application. LTP was induced in PD158780 treated slices, and the potentiated fEPSP was maintained for more than 2 hours (Tg vehicle: 108.8 ± 8.8%, n = 6, grey closed circle; WT vehicle: 146.4 ± 6.7%, n = 3, grey open circle; Tg PD158780: 154.6 ± 3.9%, *n* = 6, maroon closed circle, *P* < 0.01 compared with Tg vehicle, WT PD158780: 176.2 ± 2.8%, n = 6, maroon open circle, *P* < 0.05 compared with WT vehicle, Fig. 4A). Interrupting endogenous NRG1/ErbB signaling by PD158780 is also known to enhance the level of fEPSPs at already potentiated synapses^39^. We therefore tested whether PD158780 perfusion after LTP induction could rescue the LTP deficit in adult Tg2576. Perfusion of 10μΜ PD158780 for an hour after LTP induction increased the level of diminishing LTP and the re-potentiated fEPSPs were maintained for another 2 hours (Tg PD158780: 168.7 ± 16.1%, *n* = 6, closed circle, Fig. 4B). Therefore, disrupted NRG1/ErbB signaling might be sufficient to induce LTP in aged Tg2576 as impaired LTP was rescued in adult Tg2576 upon the perfusion of PD158780 at both HFS induction and an hour followed by the induction. In addition, we measured TPS-induced depotentiation in conjunction with 10 minutes PD158780 perfusion begun 2 minutes before HFS application. We found that LTP could not be depotentiated by the TPS application in the PD158780 treated slices (Tg vehicle: 120.2 ± 8.5%, *n* = 5, grey closed circle; Tg PD158780: 200.5 ± 30.4%, maroon closed circle, *n* = 6, *P* < 0.05, Fig. 4C) but readily depotentiated in littermate WT mice (WT: 120.6 ± 5.6%, n = 5, open circle, Fig. 4D). These data suggest that the inhibition of NRG1/ErbB signaling in adult Tg2576 is sufficient to mimic the characteristics of hippocampal synaptic plasticity in aged Tg2576.

### Deficits in memory function in aged Tg2576

**Figure 5:**
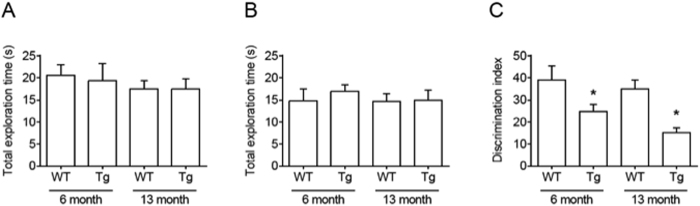
Object recognition memory in adult Tg2576. Habituation training was conducted for 3 days by exposing the animal to the experimental apparatus for 10 min per day in the absence of objects for the indicated number of days. The training session was conducted 24 h following the last habituation training. During the training session, mice were placed in the experimental apparatus in the presence of two identical objects and allowed to explore for 10 min. After a retention interval of 24 h, mice were again placed in the apparatus; however, one of the objects was replaced with a novel one. Mice were allowed to explore for 10 min. (**A**) Total exploration time in training session. (**B**) Total exploration time in test session. (**C**) Discrimination index in test session. Adult Tg2576 (Tg) mice displayed a significant reduction in object recognition memory compared with wild-type (WT) mice (24.3 vs 39.1 at 6 months, 16.7 vs 35.2 at 13 months). Data are presented as mean ± S.E.M. *P < 0.05 (n = 7–10/group, t-test).

To assess whether learning and memory function changed during ageing in Tg2576 animals, we conducted a novel object recognition memory test. In all experimental conditions, total exploration time was not different between groups (training, Fig. 5A; test, Fig. 5B). However, the discrimination index indicates impaired object recognition memory in both groups of 6 month- and 13 month-old Tg2576 mice. Novel object recognition memory performance declined progressively during aging in Tg2576 mice compared with WT littermate mice (Fig. 5C).

## Discussion

In this study, we have shown that the number of PV interneurons is significantly reduced in the hippocampus of the aged Tg2576. Critically, we reveal that NRG1/ErbB4 signaling-mediated LTP regulation, TPS-induced depotentiation and NRG1-induced LTP suppression, for which PV interneurons are essential, are impaired in the aged Tg2576. An inhibitor of the ErbB receptor prevented the LTP deficit but blocked TPS-induced depotentiation in the adult Tg2576, suggesting that the disrupted NRG1/ErbB signaling would be a strong candidate for the cause of the LTP reemergence in aged Tg2576. Our results may contribute to a potential answer to the controversy over whether LTP is inhibited^10^,^11^,^12^ or normal^13^,^14^,^15^,^16^ in the transgenic mouse model of AD (expressing APPswe mutation): LTP is only hindered for a certain period of time and then reappears in spite of concomitant behavioral deficits at that time, but the revived LTP is still abnormal due to the lack of inhibitory control and is accompanied by learning and memory deficits. Accordingly, this could account for the observed LTP found in certain AD transgenic mice^13^,^14^,^15^,^16^; LTP is indeed inducible, but the fundamental molecular mechanisms responsible for its induction and expression have actually changed. Indeed, evidence suggests that LTP impairment is associated with behavioral deficits but the degree of LTP impairment is not related to the accumulation of Aβ and age from 3 to 12 months^16^.

Among the observed changes in aged Tg2576, loss of interneurons in the AD brain has attracted many investigators’ attention to examine the physiological consequences of the cell loss and its relevance to AD. Several studies reported that GABAergic interneurons are more vulnerable to the toxic oligomeric Aβ than pyramidal neurons^23^,^40^, and are observed to degenerate in the transgenic mouse model of tauopathy^41^ or both amyloidosis and tauopathy^24^, as well as in human apolipoprotein E4 knock-in mouse^42^ and in AD patients^22^. Interneuron degeneration in the AD mouse models led to alterations in synaptic plasticity^24^,^41^, stereotypic hyperactivity^24^, inhibited sensory motor gating^41^, and impaired learning and memory^41^,^42^. In addition, recent studies have shown that both hormonal intervention and transplantation of interneuron progenitors to prevent or replace GABAergic interneuronal loss in AD mouse models could restore memory and prevent cognitive dysfunctions which are usually observed in the transgenic mice^43^,^44^. Thus, loss of interneurons is a relatively established pathological change and a direct cause of cognitive and behavioral abnormalities in AD. Our finding that PV interneurons degenerates in the hippocampus of aged Tg2576 showing abnormal synaptic plasticity also supports this notion.

On the other hand, the relationship between NRG1/ErbB signaling and AD has just started to be examined. NRG1/ErbB signaling has recently been implicated in several neuropsychiatric disorders, such as schizophrenia and bipolar disorder, because of its importance in development and function of neural circuitry, relevant to behavioral deficits and genetic association with those diseases^45^. There was also a polymorphism study suggesting that the *NRG1* gene is associated with AD^46^, (though see^47^). Some recent studies reported that, in the brains of both human and AD mouse models, expression levels of NRG1 and ErbB4 were changed^48^,^49^ and the distribution of those proteins in the hippocampus was altered along accumulated neuritic plaques^34^. However, further investigation is required to determine whether changes in NRG1 and ErbB expression actually contribute to the progression of AD. Interestingly, some studies have shown that pathophysiological features of AD can be remedied by modulating NRG1/ErbB4 signaling; treatment of NRG1 in APP/PS1 transgenic mice inhibits neuronal apoptosis in APP/PS1 transgenic mice via ErbB4-dependent PI3-kinase/Akt pathway activation^49^, and also prevents soluble Aβ_1-42_-induced LTP impairment via ErbB4 activation^50^. Collectively, therefore, although there are still only limited studies, NRG1 and ErbB kinases may play an important role in AD and other neurodegenerative disorders. In accordance with this, we also showed that NRG1/ErbB signaling was interrupted in the aged AD mouse model, as treatment of NRG1 did not suppress LTP in hippocampus of the aged Tg2576. This may be due to loss of PV interneurons, as NRG1 suppress LTP via activating ErbB4 receptor kinase in the specific neuron. We found that the percentage of PV interneurons in hippocampal CA region of aged Tg2576 was only about 70% of the percentage in adult Tg2576 and 50% in aged littermate WT. A direct relationship, however, between disruption of NRG1/ErbB4 signaling and loss of PV interneuron in aged AD mouse models, should be investigated in future studies.

Lastly, as far as we know, activity-dependent depotentiation in AD mouse model has not been previously measured. Along with LTP and long-term depression (LTD), depotentiation has been regarded as another important type of synaptic plasticity^38^. Acting as a counterbalance to the potential saturation of synaptic potentiation, depotentiation may contribute to synaptic homeostasis by which synaptic strength is maintained at a set level to be changeable by subsequent neuronal activity. In addition, depotentiation has been shown to play an important role in the refinement of developing neural circuits^51^, storage of novel spatial information^52^ and fear memory extinction in amygdala^53^. Clearly, then, depotentiation plays a critical role in normal physiological function. In this study, we observed that depotentiation could not be induced in aged Tg2576. This means that synaptic memory is still ablated in the aged Tg2576 in spite of the reemergence of LTP. Given that it has been suggested that depotentiation is related to specific neurological diseases and involves molecular mechanisms distinct from LTP and LTD^54^, it is reasonable to assume that Tg2576 would exhibit different behavioral phenotypes at specific ages when LTP and depotentiation emerge dysregulated. Indeed, previous reports suggest that aged human APP transgenic mice exhibited behavioral abnormalities such as hyperactivity^55^,^56^,^57^,^58^ and deficits in sensorimotor gating measured by prepulse inhibition (PPI)^59^, which are similar to those shown in the transgenic animals where depotentiation was not induced and NRG1/ErbB signaling impaired^30^. Such activity disturbance and PPI deficits are also observed in AD patients^60^,^61^. However, further study is needed to directly demonstrate the relationship between impaired depotentiation and the behavioral abnormalities in Tg2576 or other AD mouse models, such as the deficits in learning and memory paradigms like those we demonstrate here. This will give us further insight into the behaviorally relevant synaptic level changes occurring during the development of AD pathology. In summary, our three new findings of reduced proportion of hippocampal PV interneurons, disruption of NRG1/ErbB4 signaling and impaired depotentiation, in aged Tg2576, might shed light on the underlying pathological changes altering synaptic memory during the progress of AD.

## Methods

### Materials

Recombinant human NRG1-beta1 epidermal growth factor (EGF) domain (R&D Systems, #396-HB-050) was reconstituted in sterile PBS containing 0.1% bovine serum albumin (Sigma, #A3294). ErbB receptor kinase inhibitor PD158780 (Calbiochem, #513035) was dissolved at 10 mM in DMSO for stock solution.

### Animals

All experimental protocols were approved by the Institutional Animal Care and Use Committee of Chonnam National University. The methods were carried out in accordance with the approved guidelines. Tg2576 male mice were received from Taconic (USA, #1349) and crossbred with hybrid B6SJLF1 (from Taconic) female mice. The offspring, heterozygous transgenic and littermate wild type (WT) male mice, were used. The mice were housed in individual ventilated cages (IVC) with access to water and food ad libitum, under a 12 h light/12 h dark cycle.

### Hippocampal slice preparation

Animals were sacrificed with cervical dislocation and decapitated. The brain was rapidly removed and one hemisphere was directly placed into ice-cold artificial cerebrospinal fluid (aCSF) containing 124 mM NaCl, 3 mM KCl, 26 mM NaHCO_3_, 1.25 mM NaH_2_PO_4_, 2 mM CaCl_2_, 1 mM MgSO_4_ and 10 mM D-Glucose, perfused with gas consisting of 95% O_2_ and 5% CO_2_. The other hemisphere was used for immunohistochemistry. The hippocampus was extracted and transverse hippocampal slices (40 μm thickness) were cut using a McIllwain tissue chopper (Mickle Laboratory Engineering Co.). Following manual separation, the slices were submerged in aCSF for a minimum of 1 hour for recovery before experiments commenced.

### Electrophysiology

Hippocampal slices were placed into the recording chamber, continuously perfused with oxygenated aCSF flowing at a rate of at  ml min^−1^, maintained at a temperature of 29–30 °C. For recording, two stimulating electrode were positioned on Schaffer collateral pathway (for LTP and depotentiation input) and subiculum region (for control input) respectively, to produce field excitatory postsynaptic potentials (fEPSP). Recording glass pipettes were prepared by a micropipette puller (Sutter Instrument, P-1000) and filled with  M NaCl. The recording glass pipettes were fixed and controlled by a micromanipulator, used to locate the recording electrode to the CA1 region of the hippocampus. Following the acquisition of a stable baseline for 30 minutes, two trains of tetanus stimuli (100 Hz for 1 second with 30 seconds inter-tetanus interval) were applied to induce LTP. Depotentiation was induced with theta pulse stimulation (5 Hz for 1 minute) 5 minutes following the induction of LTP. Data acquisition and analysis was performed using WinLTP (www.winltp.com). Briefly, the slope of the evoked fEPSPs was measured, normalized to the pre-conditioning baseline and expressed as a percentage of baseline.

### Western blot analysis

Hippocampal slices were homogenized, lysed and immediately snap frozen. Protein samples were separated according to their size on sodium dodecyl sulfate (SDS)-polyacrylamide gels and transferred to PVDF membranes (Millipore, USA). The membranes were probed with primary antibodies against NRG1 (Abcam, #ab53104) and ErbB4 (Thermo Scientific Pierce, #MAI-861) overnight and then probed with secondary antibodies conjugated to horseradish peroxidase for an hour. Immunoreactive bands were detected using an ECL detection system (LAS-3000, FUJIFILM, Japan). Optical densities of the band were quantified using ImageJ software (http://rsbweb.nig.gov/ij/).

### Immunohistochemistry

Mouse hemispheric brains were fixed in 10% neutral-buffered formalin for 3 days. Brains were dissected, embedded in paraffin, and stained with hematoxylin for histopathological evaluation. Tissue blocks were sliced at a thickness of 4 μm for immunohistochemistry. Each unstained slide was stained with specific antibodies against Parvalbumin (Product No. PA1-933, 1:1000, ThermoFisher, Rockford, IL, USA) and NeuN (Product No. MAB377, 1:100, Merck Millipore, Darmstadt, Germany) using an automated immunostainer (Bond-maX DC2002, Leica Biosystems, Bannockburn, IL, USA). Programmed heat-induced epitope retrieval was carried out using bond epitope retrieval solution 1 (containing Tris EDTA, pH 9.0) for Parvalbumin antibody and bond epitope retrieval solution 2 (containing Ethylenediaminetetraacetic acid (EDTA) buffer at pH 9.0) for NeuN antibody. Negative controls were processed similarly in the absence of primary antibodies.

### Object recognition memory test

The experimental apparatus consisted of a black polyvinyl plastic square open field (45 cm × 45 cm × 45 cm). Habituation training was conducted for 3 days by exposing the animal to the experimental apparatus for 10 min per day in the absence of objects for the indicated number of days. The training session was conducted 24 h following the last habituation training. During the training session, mice were placed in the experimental apparatus in the presence of two identical objects and allowed to explore for 10 min. After a retention interval of 24 h, mice were again placed in the apparatus; however, one of the objects was replaced with a novel one. Mice were allowed to explore for 10 min. The objects chosen for this experiment included a plastic orange and wooden trigonal prism, both approximately the same height. The durations of time mice spent exploring each object (familiar object, *T*_*familiar*_; novel object, *T*_*novel*_) were recorded. The discrimination index was calculated by following formula: (*T*_*novel*_ − *T*_*familiar*_)/(*T*_*novel*_ + *T*_*familiar*_) × 100.

### Data analysis

Data were analyzed from one slice per mouse (n = number of slices = number of mice). In electrophysiology, the fEPSP slope was expressed as the mean ± SEM (standard error of the mean) and relative to a normalized baseline. For the comparison of LTP and depotentiation between groups, fEPSPs at the end of recording were used. An unpaired two-tailed Student’s t-test was used for the statistical analysis of the electrophysiology data and novel object recognition tests, and two-way ANOVA and Turkey’s multiple comparison tests for the Western blotting and immunohistochemistry data. All statistical calculations were performed using GraphPad Prism (GraphPad Software Inc.).

## Additional Information

**How to cite this article**: Huh, S. *et al*. The reemergence of long-term potentiation in aged Alzheimer’s disease mouse model. *Sci. Rep.* **6**, 29152; doi: 10.1038/srep29152 (2016).
